# Primary Cutaneous CD30+ Anaplastic Large Cell Lymphoma: A Rare Association With Large Plaque Parapsoriasis

**DOI:** 10.7759/cureus.11228

**Published:** 2020-10-28

**Authors:** Ionela Manole, Alexandra-Irina Butacu, Iulia-Elena Negulet, Marius-Cristian Valcoci, George-Sorin Tiplica

**Affiliations:** 1 Dermatology, Carol Davila University of Medicine and Pharmacy, Bucharest, ROU; 2 Dermatology, Colentina Clinical Hospital, Bucharest, ROU

**Keywords:** cd30+ anaplastic large cell lymphoma, large plaque parapsoriasis, cutaneous t-cell lymphoma

## Abstract

Primary cutaneous CD30-positive lymphoproliferative disorders represent the second most common subgroup of cutaneous T-cell lymphomas and include lymphomatoid papulosis, primary cutaneous anaplastic large cell lymphoma, and borderline lesions. Primary cutaneous anaplastic large cell lymphoma is characterized by the presence of solitary or localized nodules or tumors located on the extremities or the cephalic or cervical region. Large plaque parapsoriasis is a chronic inflammatory disorder that associates a high risk of progression to mycosis fungoides. We report a case of CD30+ primary cutaneous anaplastic large cell lymphoma in a patient with a long history of large plaque parapsoriasis.

## Introduction

Peripheral T-cell lymphomas encompass 15% of non-Hodgkin lymphomas in adults [1]. Primary cutaneous CD30-positive lymphoproliferative disorders represent the second most common subgroup of cutaneous T-cell lymphomas after mycosis fungoides and include lymphomatoid papulosis and primary cutaneous anaplastic large cell lymphoma [2]. These three clinical entities present similar histopathological findings and are differentiated based on clinical findings and evolution of lesions.

Lymphomatoid papulosis consists of a recurrent papulonodular, hemorrhagic, or necrotic cutaneous skin eruption with spontaneous resolution over the course of a few weeks [3]. Primary cutaneous anaplastic large cell lymphoma is characterized by the appearance of a single lesion, usually as a solitary ulcerated tumor or as grouped nodules, located on the extremities or the cephalic or cervical region. Spontaneous resolution is rare and approximately 10% of patients present with extracutaneous involvement [4].

Large plaque parapsoriasis is a chronic inflammatory disorder characterized by the presence of large erythematous plaques covered by fine scales, generally located on sun-protected areas. Large plaque parapsoriasis affects mostly middle-aged or older individuals, with approximately two-thirds of patients being males [5]. Due to the high risk of progression to mycosis fungoides, cases of large plaque parapsoriasis require close clinical monitoring. Differentiating large plaque parapsoriasis from early mycosis fungoides may be difficult and may require several histopathological examinations before having a definitive diagnosis. Even though large plaque parapsoriasis can progress into mycosis fungoides, its association with C30-positive anaplastic large cell lymphoma has rarely been described in the medical literature. Herein, we present a case of a C30-positive anaplastic large cell lymphoma in a patient with a long history of large plaque parapsoriasis, arising after a local trauma.

## Case presentation

**Figure 1:**
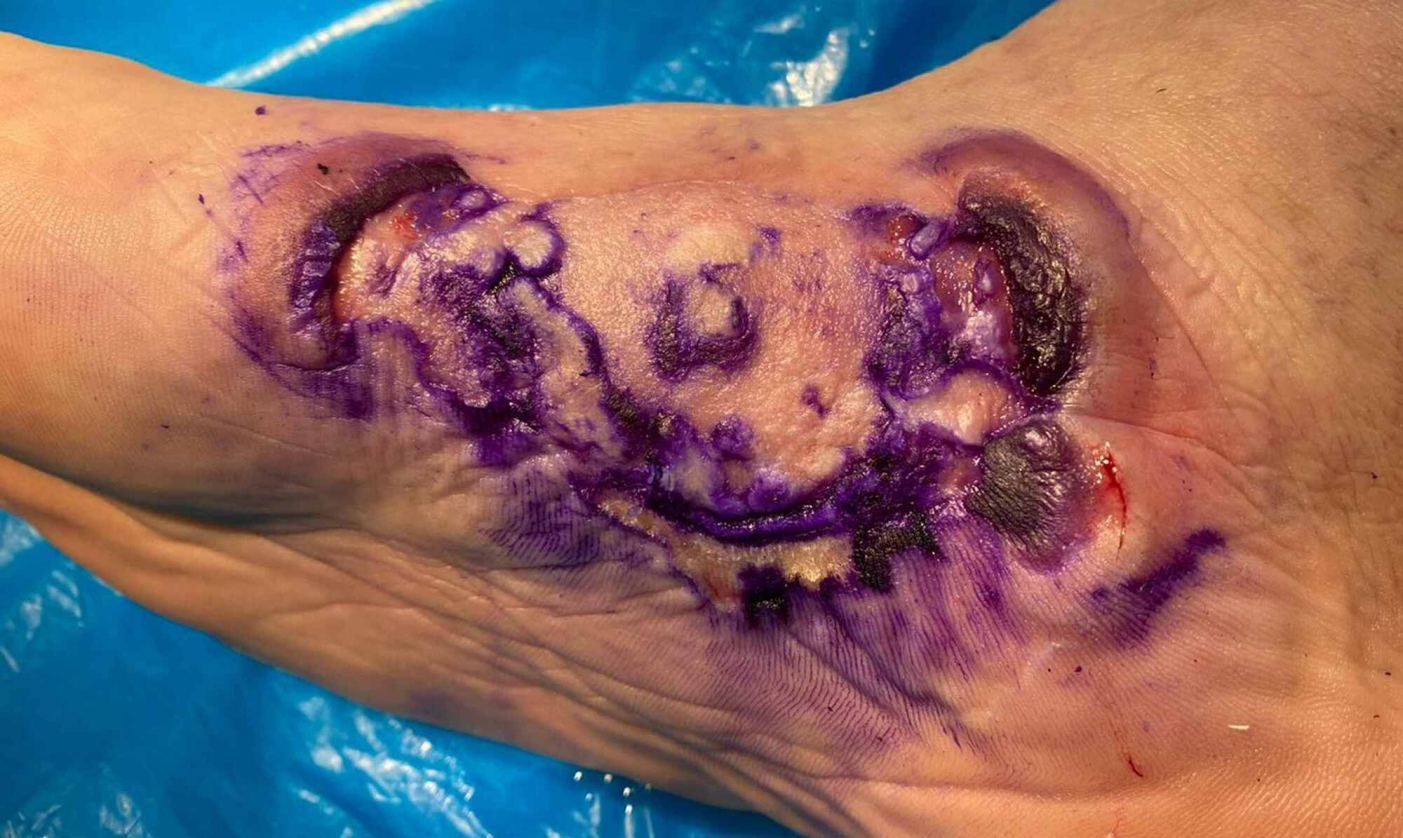
Erythematous ulcerated placard surrounded by violaceous raised borders

A 51-year-old male presented for consultation with an erythematous ulcerated placard located on the inner side of the right foot. The lesion appeared three months prior following immobilization with a plaster cast for medial malleolus fracture and was unsuccessfully treated with topical antiseptics. On clinical examination, the lesion measured 15 x 8 centimeters and was surrounded by violaceous raised borders and tendency to central clearing (Figure 1).

**Figure 2:**
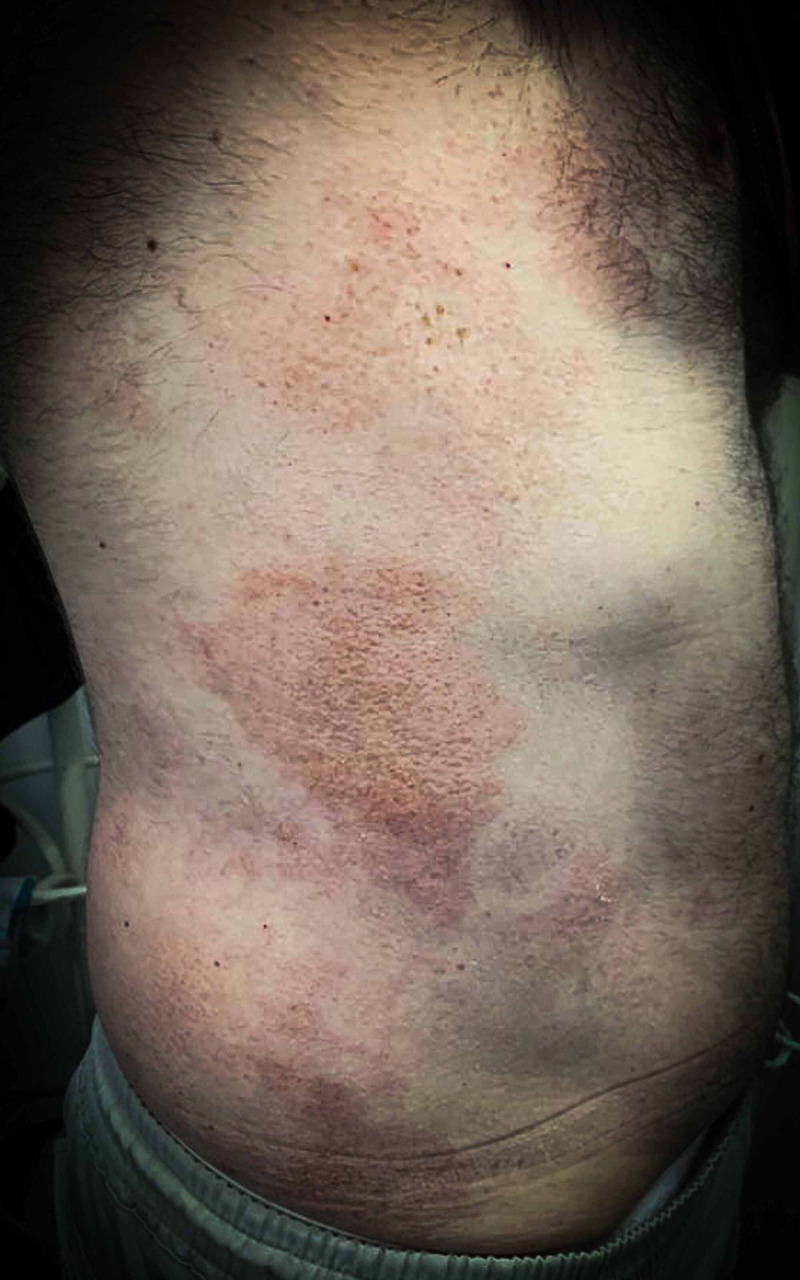
Erythematous placards, covered by fine white scales, on the right latero-thoracic area

Furthermore, the clinical examination also revealed numerous erythematous, slightly pruritic placards, irregularly shaped, on the lateral sides of the trunk. The most representative lesion measured 15 centimeters in the diameter, was covered by fine white scales, and was located on the right laterothoracic area (Figure 2).

The patient has been diagnosed three years prior with large plaque parapsoriasis, which was confirmed through histopathological examination, with episodes of periodic flares and remissions under local treatment with corticosteroids. Personal medical history also included hypertension, chronic hepatitis B, chronic hepatitis C, and autoimmune thyroiditis.

The patient denied any systemic symptoms such as fever, chills, unintentional weight loss, or night sweats. Physical examination was unremarkable for peripheral lymphadenopathy or hepatosplenomegaly.

Laboratory investigations revealed monocytosis (monocyte count of 1,120/microL [13.4%]), without any other hematological abnormalities such as anemia, thrombocytopenia, or leukocytopenia. The bacteriological examination of the ulcerated lesion showed the presence of *Pseudomonas* spp. sensitive to ciprofloxacin and ceftazidime. Furthermore, the thoracoabdominopelvic CT did not detect any adenomegaly or organomegaly.

A lesion skin biopsy was performed, showing a diffuse, malignant, lymphoid tumoral proliferation with large cells with rounded vesicular nuclei and basophilic cytoplasm. The tumoral cells were arranged in compact islands and also in focal clusters surrounded by small lymphocytes, eosinophils, and Langerhans histiocytes. Immunohistochemical studies revealed the immunophenotype of the neoplastic lymphocytes as CD3+ and CD30+ with cytotoxic protein expression (Granzyme B). CD20, epithelial membrane antigen (EMA), and anaplastic lymphoma kinase (ALK) were negative.

The patient was referred to the Hematology Department where a bone marrow biopsy was performed without identifying any pathological changes.

The diagnosis of CD30+ primary cutaneous anaplastic large cell lymphoma was established, and a planned regimen treatment of 44-46 Gy in 22-23 fractions of radiation therapy was initiated.

**Figure 3:**
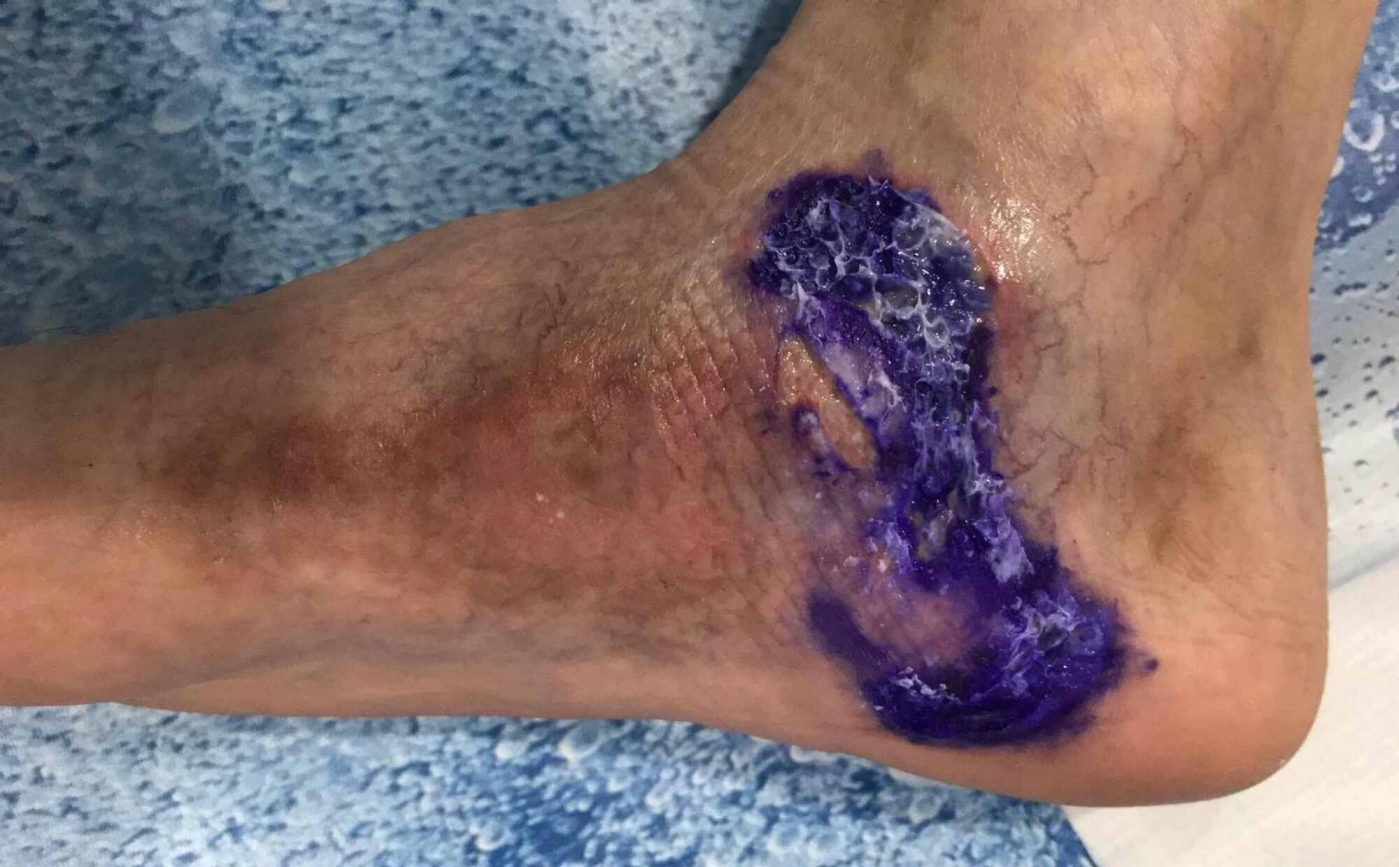
Clinical aspect following seven fraction of radiation therapy revealing central clearing of the ulcerated lesion

Follow-up after seven fractions of radiation therapy revealed a favorable outcome, with a significant decrease in the diameter of the ulcerated lesion (Figure 3).

## Discussion

Primary cutaneous anaplastic large cell lymphomas are usually solitary tumors or grouped or multifocal nodules located on the upper half of the body in middle-aged men [6]. Primary cutaneous anaplastic large cell lymphomas should be differentiated from secondary cutaneous anaplastic large cell lymphomas in which systemic involvement is present and skin is the most common extranodal site [7]. Clinical and histopathological features may overlap between primary cutaneous CD30-positive lymphoproliferative disorders as and other systemic and cutaneous lymphomas; therefore clinicohistopathological and immunohistochemical studies are extremely important when establishing diagnosis [8].

Primary cutaneous anaplastic large cell lymphoma associates favorable prognosis with a 95% survival at 10 years when extracutaneous involvement is not present and a 76%-96% survival rate at 5 years when more than one nodal basin is affected [9]. Favorable prognostic indicators include age below 60 and spontaneous resolution of lesions, whereas systemic findings and extensive limb involvement are associated with poor prognosis [10].

From a clinical point of view, as seen in this case presentation, primary cutaneous anaplastic large cell lymphoma usually presents as a rapidly growing solitary tumor larger than 2 cm in diameter, which may ulcerate. Even though the rapid evolution of lesions may be alarming, patients do not associate systemic findings similar to those found in systemic lymphomas, such as weight loss, fever, or night sweats [11]. Spontaneous resolution may appear after three to four weeks in approximately 28% of cases, even though lesions may reoccur. Extracutaneous disease is rare, reported in approximately 13% of cases [12].

Laboratory investigations include histopathological examination, which reveals nodular dermal infiltrates of sheets of atypical large anaplastic lymphocytes, consisting of cells with irregular horseshoe‐shaped eosinophilic nuclei, an abundant cytoplasm, and an ulcerated epidermis [13]. Immunohistochemical studies show the presence of CD30 in more than 75% of tumor cells, without the presence of a cytotoxic phenotype. ALK, usually associated with systemic anaplastic lymphomas, is not routinely observed in patients with primary cutaneous anaplastic forms [6].

Treatment of primary cutaneous anaplastic large cell lymphoma consists of surgical excision or local radiotherapy with favorable outcome [14].

In this case presentation, considering the erythematous plaques located on the trunk, development of the placard on the inner side of the foot also raised the suspicion of progression of large plaque parapsoriasis into cutaneous T-cell lymphomas’ tumor stage. This hypothesis was not supported by the clinical evolution of lesions, as the erythematous placards located on the latero-throacic areas completely disappeared after a short course of topical steroids. Additionally, the solitary ulcerated lesion appeared on the inner foot region, an area not characteristic for mycosis fungoides. Additionally, the solitary ulcerated lesion appeared on normal-looking skin and not on a pre-existing lesion of mycosis fungoides.

Furthermore, the histopathological examination revealed anaplastic cells with irregular nuclei and basophilic cytoplasm and did not identify the characteristic cerebriform nuclei or band-like lymphocytic infiltrate, as found in cases of mycosis fungoides [15].

Also, the favorable evolution of the ulcerated lesion after a course of seven fractions of local radiotherapy reinforced the diagnosis of primary cutaneous anaplastic large cell lymphoma.

Nonetheless, the association between large plaque parapsoriasis and primary cutaneous anaplastic large cell lymphoma is rare, even though it has been cited in medical literature [16], and patients should be monitored in order to diagnose the evolution of parapsoriasis into mycosis fungoides at an early stage.

Additionally, another significant aspect to be considered is the occurrence of a cutaneous T-cell lymphoma after a recent trauma. Medical literature presents cases of mycosis fungoides in patients who reported trauma remotely (years before the skin lesion appeared), but no patient had recent trauma [17]. Also, it is difficult to establish whether the cutaneous T-cell lymphoma on the foot was a result of the local trauma or was associated with the large plaque parapsoriasis, even though large plaque parapsoriasis can progress into cutaneous T-cell lymphomas on the same lesion and not elsewhere in the body.

Furthermore, the use of a cast that may contain carcinogenic materials [18] might be a etiopathogenic factor which was involved in the appearance of the cutaneous large cell CD30-positive lymphoma, making the hypothesis of its association with large plaque parapsoriasis less probable.

In other words, cutaneous T-cell lymphomas can occur in other locations than large plaque parapsoriasis in patients already having it, likely due to underlying immunodeficiencies.

## Conclusions

Primary cutaneous CD30+ lymphoproliferative disorders should always be differentiated from other cutaneous or systemic lymphomas, including mycosis fungoides, as it represents a low-grade malignant lymphocytic proliferation with favorable prognosis and possible spontaneous resolution.

Close follow-up of patients diagnosed with large plaque parapsoriasis is of utmost importance, as the evolution of lesions into mycosis fungoides may appear rapidly and be associated with an aggressive evolution of this affliction with a less favorable outcome.
